# Making modafinil: Classification and serendipity in drug development

**DOI:** 10.1177/03063127251322113

**Published:** 2025-03-18

**Authors:** Stephen J Scholte, Ohid Yaqub

**Affiliations:** Science Policy Research Unit (SPRU), University of Sussex, Brighton, East Sussex, UK

**Keywords:** pharmaceutical innovation, drug classification, modafinil, drug development, bounded serendipity

## Abstract

How does a compound become a drug, and how do we decide for whom the drug is intended? Building a history of modafinil, this article examines how classification and serendipity affect drug development. We explore how mental health categories interact with drug development by tracing: how compound CRL40,476 was inadvertently created while exploring other compounds, and then became a focal point for development efforts; and how it secured Schedule IV status (low potential for abuse), orphan drug status (for niche markets), and then blockbuster drug status (>$1bn in annual sales). Classification of modafinil and its uses were negotiated under conditions of uncertainty, requiring substantial efforts to align interests across a wide array of institutions. We highlight these contingencies to show the considerable efforts that go into finding, and creating, markets for drug development. Taking these efforts for granted may confuse invention with innovation and is likely to lead to understatement of the costs and choices involved in drug development, particularly where mental health categories are concerned.

Since it was incidentally created 50 years ago, in the metabolic system of a rodent, little about the compound that would eventually become modafinil has remained stable. The compound has at various points in its journey been termed a psychostimulant, a wakefulness promotion agent, and a smart drug. It has been the intellectual property of a small, family-owned, pharmaceutical firm, the headline drug for a large multinational, and a generic to be made by companies around the world. It has been an orphan drug intended for a small population of narcolepsy patients, and a blockbuster drug making over a billion US dollars annually. It has been described as entirely safe and without side effects, and had marketing approval revoked because of safety concerns. This case study—part history, part historiography—traces the emergence of modafinil from the initial patents for adrafinil in 1976 up until the controversies over the scope of its usage in the 2000s. This article complements existing studies on pharmaceutical use by going further upstream and examining their innovation process (Kumar, 2008).

A short version of modafinil’s development highlights the role of good fortune: Researchers were looking for an analgesic and were surprised instead to find a narcolepsy drug. The researchers themselves described it as ‘a serendipitous discovery’ (Rambert et al., 2007), invoking the notion of unexpected and beneficial discovery (Yaqub, 2018). It was an unexpected gift and, given the array of uses the drug would eventually be put to, it was a gift that kept on giving. One of its approved uses, shift work sleep disorder (SWSD), was not even included in standard disorder classifications when modafinil was created.

Examining what counts as serendipity is useful for investigating how categorization interacts with drug development; the story is more complex than simple serendipity may imply. We show that the eventual use of modafinil for narcolepsy may have been unexpected, but it was not completely random. We parse the connections between modafinil and the diagnostic categories designated as approved uses (i.e., drug indications), from the initially vague and often varied descriptions in patent documents to the final specific uses listed on drug labels. We also trace its regulatory categories, which indicate potential for drug abuse (i.e., scheduling), and policy categories, which offer market privileges (i.e., market approval, orphan status and patent protection). These categories shape what studies are done on a compound, how a compound becomes a drug, and, ultimately, who is affected by the drug.

## Framework and methods

The entry point for this case study was an abstract chemical structure. To call it a drug is to pre-empt the outcome. An approach that is agnostic to outcomes highlights the limited extent to which the possible uses for a compound can be determined by its physical and chemical composition alone. To link the inherent properties of a compound to its eventual uses is far more difficult than is widely appreciated. Indeed, even the term *drug discovery* may be misleading, as if drugs are merely waiting to be discovered and selected *off-the-shelf*. A compound is not a drug until its indication is established.

In following the evolution of this case, we drew broadly on a Latourian framework, offering ‘a way of starting inquiry on the basis of uncertainty’ (Gad & Jensen, 2010, p. 63; see also, Sismondo, 2018). We take from Latour (1986, 1988) an understanding of facts, which in this context pertains to perceptions of the natural and social character of modafinil. A retrospective focus on the natural character of a drug may provide a veneer of inevitability and predictability about its eventual uses. Similarly, a retrospective focus on the social character of a drug may create an illusion of choice without constraints about its eventual uses. However, we do not assume that the distinction between the natural and social character of modafinil is obvious or fixed. Rather, the distinction emerges from work to negotiate these boundaries. Perceptions of natural and social boundaries change as caches of references and allies are built through negotiations, or ‘trials of strength’ (Latour, 1988). These are dynamic interactions between different components of the system for turning a compound into a drug.

This approach allowed us to explore unexpected and beneficial discovery as a constrained process, which we term *bounded serendipity.* Our focal points are instances in which the ambiguity in the characteristics and identity of what is to become the drug modafinil were visible in our source material. Latour emphasizes the work that is done to create apparent immutability; ‘real worlds’ are the ‘consequence of lines of stable force and not the cause of their stabilization’ (Latour, 1988, p. 228). Our case begins at a point of relative ‘irreduction’ (Latour, 1988, p. 158): The existence of the compound we shall follow has not yet been noted by humans, or tested, or spoken for. The various firms that have owned the compound were the instigators of many trials by which they attempted to enlist the voices of a host of varied actors to speak on behalf of the compound. With each subsequent codification in a patent, a scientific publication, or a regulatory document, the compound gained further potency and another ally. In each instance, the outcome was not a given. When successful, these actors are in alignment and their voices appear in unison. The narrowing uncertainty reached a closure (MacKenzie & Wajcman, 1999), where the provisional state of knowledge (about a candidate) became a stabilized artefact (an approved drug). Closure could be revisited, in our context, as drug repurposing.

This approach is well suited for studying the uncertainty inherent in drug discovery and development. Most attempts to produce a drug fail. That is, most drug candidates do not make it to market, nor even to clinical trial phases where they are tested in humans (Waring et al., 2015). Understanding the nature of these uncertainties is not a trivial matter. Rather it goes to the heart of how drugs are developed, regulated, and marketed. Moreover, the remarkable inability to foresee clearly the uses to which a drug candidate will be put reflects just how much work goes on in research and development. This remains the case even in the latter stages of a process we characterize as bounded exploration, where the breadth of the search space is constrained and narrowing. This is because there is uncertainty not only about the potential effects of compounds, but also about how these effects can be combined with regulatory, diagnostic and policy categorization. From this, we can see that most of what is termed research and development is development.

The discovery phase of pharmaceutical innovation is largely insulated from the complexities of the external world. Testing proceeds under simplified laboratory conditions or in animal models (Yaqub & Nightingale, 2012). Drug discovery trajectories involving compound synthesis, phenotypic drug discovery and resource-intensive screening processes are often presented as precision medicine or rational drug discovery (Gittelman, 2016; Nightingale, 2000). This contrasts with serendipitous drug discovery, in which an unexpected connection between a compound and the physiological, behavioural, or phenomenological response is observed (Thompson & Copeland, 2023). This can occur when mechanisms are poorly understood, and even when the compound is a long way from being viewed as a drug. Additional forms of drug discovery include methods that draw on natural substances or existing treatments and seek to refine and/or expand the scope of these compounds (Greenblatt et al., 2023; Mathur & Hoskins, 2017). From a distance a particular discovery may appear to neatly fit one of these models, but a more detailed examination tends to suggest high levels of overlap.

During the development of psychopharmacology, many (if not all) of the breakthroughs in the field are said to have been due to serendipity (Klein, 2008). Characterizing an observation as serendipitous requires that it leads to an outcome deemed beneficial, or that it be a solution to a problem. This is only possible retrospectively. For a drug to count as a solution it must have been aligned with a problem; in this case a diagnostic category and the actors on behalf of which this category is seen to speak. It must also have passed through numerous trials posed by institutional actors, safety and efficacy tests, and applications for patent protection. This development phase is often marked by testing in humans. Moving from animal testing to clinical testing resembles a leap into uncertainty, where concerns become conspicuously framed in terms of safety and efficacy (Yaqub, 2017). Safety and efficacy each have their own categories, whose boundaries become contested in negotiations over drug scheduling and progression through formal clinical trial phases and approvals for markets.

It is in this sense that approaching drug development as an often serendipitous and technological process, as opposed to a scientific one, can offer some insights. While Latour would reject offhand a bifurcation of science and technology, the distinction offered by Nightingale (2004)—based on orientation towards different kinds of goals—can help the interpretation of the actions of the firms driving the trajectory of our case. Pharmaceutical firms employ many of the tools and techniques that fit familiar descriptions of science, but their end goals are not the production of knowledge, nor the assignment of a particular diagnostic category. These are merely steps in a flexible process towards an approved use for a compound. The ultimate goal is to produce a marketable drug with exclusive rights for as long as possible.

Within psychopharmacology, further uncertainty ensues from the nature of the diagnostic categories themselves (Kendler et al., 2011; Pickersgill, 2011). Despite a century of attempts to pin down neat categories and physiological etiologies, psychiatric disorder categories remain heterogenous and overlapping (Conway et al., 2022). Moreover, they feature ‘dynamic nominalism’ (Hacking, 1995), whereby labelling people with categories can shape their self-image, their actions, and their social and institutional environments. People who are categorized, change with categorization and, in turn, change the category. Where the framings of categories are particularly slippery and ill-defined, we might expect category-crossing to be more frequent.

Methodologically, we followed chains of reference (Latour, 1986). Our attention was drawn to modafinil initially because one of its approved uses, shift work sleep disorder (SWSD), was not even included in standard disorder classifications when modafinil was created. We began with two parallel lines of inquiry: on the serendipitous nature of modafinil, and, on how its links with SWSD were established. From these starting points we allowed the case to branch out into many avenues of uncertainty that appeared in the literature, such as how its links with other approved and non-approved uses were established. The search process was thus iterative and varied in topic and method. Sources that indicated uncertainty or contestation were flagged for further inquiry. This led us both backwards and forwards chronologically through the literature, drawing on citations in the documents themselves and ‘cited by’ tools in major databases such as SCOPUS, Google Scholar, and Web of Science.

Initial searches were focused on establishing a research trajectory leading up to, and shortly following, the initial patents for modafinil’s predecessor, adrafinil, using various patent databases—WIPO Patentscope, Google Patents, Espacenet, and national patent databases—alongside academic databases such as SCOPUS and Web of Science. Relationships between the compounds and their potential diagnostic indications led us through various iterations of diagnostic manuals, ICD, DSM and the International Classification of Sleep Disorders (and its predecessor, along with the workshop and conference proceedings which predate this). We examined academic literature studying various diagnostics, symptoms and treatment, and compared this to the reported effects of adrafinil and modafinil in academic literature, clinical trials and patents.

We searched for further information on each of the people who play a major role in modafinil’s trajectory. To build a picture of these individuals and the context within which they acted, we searched for their names in sources such as government proceedings, conference publications, publication records, bibliographies, interviews in trade magazines or news media. Numerous attempts to arrange interviews with those still alive were deflected or outright rejected.

The US Food and Drug Administration (FDA) provided us with extensive primary documentation on the negotiations about scheduling and labelling, alongside hints at potential future indications and market manoeuvres in their approval package documentation (FDA, 1998). We further searched for relevant material in government records (e.g., UK parliamentary and US Congressional records), European, US, and UK regulatory agencies’ websites (EMA, FDA, and MHRA), clinical trials registries (clinicaltrials.gov, clinicaltrialsregister.eu, trialsearch.who.int) legal proceedings (via the PACER database, Casemine, and various state specific court record depositories), and marketing materials (largely found on archived versions of the modafinil website using the Wayback Machine). These searches were targeted towards specific moments in modafinil’s trajectory through combining search terms and/or date ranges.

Drawing on these varied sources, a corpus of 919 texts was compiled over several years, scanning abstracts, following references, and renewing searches until data saturation led to marginal payoff from any further data collection. A subset of these sources was used for critical close-reading: 241 research articles; 26 patents (excluding duplicates); 4 books of conference abstracts; 29 government reports, along with dozens of guidelines to physicians and pharmaceutical firms; two complete new drug approval packages and seven warning letters from the FDA; memoranda and court reports covering 6 cases in depth; 21 press releases from the FDA, MHRA, and EMA; and a host of newspaper and magazine articles. The publication dates range from 1971 to 2023. With our selected texts, we built a single-context case-study with two embedded units of analysis: compounds and categories. We followed not just the eventual chosen drug candidate but also dropped candidates, and their changing fit with a range of diagnostic, regulatory and policy categories.

## Empirical findings

### Adrafinil and the antecedents of modafinil

Modafinil was, in a manner of speaking, created before it was discovered. As the primary metabolite of adrafinil, modafinil is likely to have first existed inside the rodent who was the first test subject^1^ for adrafinil. Modafinil probably never existed in nature, and likely never existed in a laboratory until investigations into adrafinil began.

Adrafinil is a synthetic compound identified by two chemists in 1974 (Billiard & Broughton, 2018). The earliest documentation regarding adrafinil is in patents filed in 1975 and 1976 in the name of Laboratoire Lafon, for a family of benzhydrylsulphinyl derivatives which may be ‘useful in therapy for treating disturbances of the central nervous system’ (Lafon, 1978, p. 1). Lafon sought patent protection for nine compound structures and their preparation methods in France, Germany, Canada, the US, and the UK.

From the family of nine compounds, one was afforded prominence: CRL40,028 (later named adrafinil). With adrafinil,^2^ ‘one is dealing with a psychostimulant’ (Lafon, 1978, p. 8). Compared to established stimulants, Lafon claimed that adrafinil’s effects are more akin to caffeine than to amphetamine-type compounds. In mice and rats, adrafinil led to ‘excitation and hyper-reactivity’ (Lafon, 1978, p. 7), increased motility and, at higher dosages, increased willingness to endure an electric shock, alongside a ‘virtual absence of specific toxicity’ (Lafon 1978, p. 8). For the behavioural tests, adrafinil was given its own section, whereas the tests for the rest of the family of compounds described were lumped together under one heading ‘Tests on the other products’. Three were mentioned as increasing motility, but also as carrying with them ‘stereotypies brought about by amphetamine’ (Lafon, 1978, p. 8), two appeared to have sedative effects, one was described as having effects similar to existing tricyclic antidepressants, and one was described as having an ‘anti-oedema effect’. The nine compounds produced a wide range of effects but were structurally similar enough to all be ‘benzhydrylsulphinyl derivatives’; the macro effects of micro changes in compound structure are both constitutive and indicative of the difficulties in predicting the outcomes of psychopharmacological innovation (Nightingale, 2004).

The target for Lafon’s chemists was the development of novel analgesics (Billiard & Broughton, 2018). This finds some congruence with the research trajectory documented in Lafon’s patent history, which indicates previous success in producing compounds that contained enough potential as analgesics to motivate seeking patent protection. In this sense, the discovery of adrafinil could be considered serendipitous, that is, a targeted search for an analgesic that led instead to the discovery of a psychostimulant. (There are also patents claiming treatment for hypertension, cardiac arrythmia, and even use as a shampoo ingredient, amongst other things.) The element of unexpectedness is further emphasized by the limited effort to explain how the apparent effects of the compounds might have come about. There was no mention of a mechanism of action.

However, this sense of unexpectedness may have been tempered by comparison with other drugs, which provide hints to those ‘skilled in the art’. If a compound acts upon a given receptor site, it is likely to mimic the effects of other drugs known to act upon the same receptor site. After the patents were filed, Lafon employees published a paper outlining a ‘possible alpha-adrenergic mechanism’ for the hyperactivity induced by CRL40,028 (Duteil et al., 1979), working from a point of comparison with existing stimulants.

There was no mention of analgesics in the benzhydrylsulphinyl patents, nor description of any testing to determine the compounds’ analgesic potential. At this stage, our case study is actually of a series of compounds, one of which is given preferential treatment in a patent application and produces interesting effects when injected into mice and rats. The compounds were ‘said to be effective in the treatment of disorders of the central nervous system’ (Lafon, 1978). The patents represent a point in time at which the trajectory of adrafinil and the related family of compounds has already shifted, from hypothesized analgesic to a new, vague functional space, relating to the central nervous system but without specificity in the target diagnostic category for eventual use. Negotiations to mitigate this uncertainty are reflected in the comparisons made to existing anti-depressants and stimulants (amongst others) in the patents. Connecting an unexpected discovery to a specific need would take more work; one cannot market a drug for general ‘disorders of the central nervous system’.

To establish the necessary connection between compound and indication requires moving out of the laboratory. In the move to the clinic, the individual given most credit is Michel Jouvet (see Bastuji, 2018; Billiard & Broughton, 2018; Lin et al., 2018). A neurophysiologist and a clinician, Jouvet is widely cited for his work on paradoxical sleep (REM sleep). At the time he became involved with adrafinil/modafinil, Jouvet was a professor and director of a department of experimental medicine in Lyon, and a director at the French Institute of Health and Medical Research. From 1977, he was also a member of the Académie des Sciences.

It is to show the strength of the ally Lafon was about to make that we have outlined Jouvet’s biography. As a friend of Louis Lafon (Lin et al., 2018), Jouvet was given samples of adrafinil to test. While Jouvet was testing adrafinil on cats in his laboratory, other researchers had also received samples and were busy watching the responses of rhesus monkeys to adrafinil (Milhaud & Klein, 1985). Both laboratories observed stimulant-like effects but without the side-effects associated with other known stimulants (Billiard & Broughton, 2018).

Adrafinil trials in humans appear to have begun at Jouvet’s behest at the Neurological Hospital in Lyon around 1978. The compound produced inconsistent results. Bastuji (2018, p. 75), Jouvet’s co-author and colleague in the clinic at the time, wrote that adrafinil was ‘clinically very well tolerated but had little effect on vigilance despite our giving up to six tablets a day to try and obtain a significant effect on excessive daytime sleepiness’. The patients were individuals diagnosed with narcolepsy or idiopathic hypersomnia.

Adrafinil was approved for use in France in 1981,^3^ to treat deficits in alertness and cognitive decline in aged patients, under various similar terms. How adrafinil moved from Jouvet’s experimental use in people diagnosed with narcolepsy or the very broad, symptom-based category of idiopathic hypersomnia, to use in a particular sub-group of elderly people is somewhat opaque. The motivation for directing the drug toward this group of patients (tied together not through specific diagnostics, but rather symptomatology) was suggested by Billiard and Broughton (2018): The pharmaceutical firm Sandoz (now part of Novartis) was seeking a replacement for its drug Hydergine, which was used to improve elderly patients’ cognitive and self-care abilities. This suggests a market-driven move to narrow the scope of the target population for adrafinil. Billiard and Broughton (2018) also noted that Lafon, unlike the larger Sandoz, did not have the resources to fund clinical trials at that time, yet a passable application was put together.

Use with narcoleptic patients, as Jouvet had begun some years previously, became formally sanctioned in 1985 when adrafinil was approved for this indication in France. Adrafinil was never granted approval in the US or the EEC (or later the EU) and, in 2011, following a safety and efficacy review, the French regulator withdrew adrafinil’s approval. Since then, it has been regularly referred to as a nootropic or cognitive enhancer and is mentioned on many ‘biohacking’ websites. It has been studied as a treatment for behavioural problems in aged canines, which led to US Patent 6180678. A 2017 application to have adrafinil accepted as a ‘new dietary ingredient’ was rejected by the FDA, stating that there was ‘inadequate information to provide reasonable assurance that such ingredient does not present a significant or unreasonable risk of illness or injury’ (FDA, 2017, p. 2). Beyond this, adrafinil’s fortunes appear to have withered.

### Modafinil takes centre-stage

Lafon and Jouvet turned their attention from adrafinil to its primary metabolite, modafinil (i.e., modafinil is one of the compounds into which mammals’ metabolic systems convert adrafinil). In December 1979, US Patent 4177290 was granted to Lafon for producing ‘novel acetamide derivatives … discovered to have useful pharmaceutical activity on the central nervous system’ (Lafon, 1979, p. 1). The stimulant effects of these derivatives were noted but no claims were made that they could replace particular existing treatments. The compound first and most thoroughly presented was CRL40,476 (later named modafinil).

The patent described results from animal trials using modafinil. Mice were trained, through the use of electric shocks, not to cross a given surface. Administering modafinil led to ‘an increase in the number of punished passages (probably in connection with an exciting effect) … [and] … it counteracted the convulsing effect of electric shock’ (Lafon, 1979, p. 5). Higher doses led to resumption of the conditioned avoidance response, alongside stereotypies associated with amphetamines. At extremely high doses, modafinil led to reduced motility, breathing difficulty, and abnormal gait, and, within 24 hours, death.

The patent mentioned informal trials in humans, but only at the end of the document and only in brief and vague terms: two sentences about the ‘very favourable’ results achieved treating ‘aged asthenics’ and ‘neuroleptics’ (Lafon, 1979, p. 6). Similar to the patents for adrafinil, modafinil was also described as a psychostimulant, and comparisons to existing stimulants were again noted. Similarities to the antidepressant imipramine were also noted in the patent. However, the ambiguity in its function was still evident.

The primacy of modafinil over the other compounds was not inevitable at the time. There were at least six other Lafon patents within a three-year interval, covering variations on the same family of compounds.^4^ Moreover, a 2018 article published by some of the remaining researchers in Jouvet’s laboratory reveals a degree of unexpectedness in the results reported in the patents (Lin et al., 2018). The mice ‘were so quiet that modafinil was thought at the beginning to be an inactive metabolite of adrafinil and even a “good sedative”’ (Lin et al., 2018, p. 40). Given that the original documentation on adrafinil notes that ‘at no dose does CRL40,028 bring about a major decrease in motility’ (Lafon, 1978, p. 7), this finding would indeed have been unexpected. Jouvet’s laboratory, with its history of sleep research and sleep-tracking instrumentation, became a powerful ally for the newly synthesized compound. After entreaties from Louis Lafon, the compound was taken in by Jouvet’s laboratory and administered to a cat, who was expected to fall asleep. It did not. The cat remained awake throughout the night, ‘without even one minute of sleep’ (Lin et al., 2018, p. 41).

Although Bastuji (2018, p. 74) acknowledges Jouvet’s role in ‘the discovery of the stimulant effect of modafinil’, Billiard and Broughton put greater emphasis on Jouvet’s efforts to develop it as a treatment for narcolepsy. Jouvet is reported to have begun prescribing modafinil to patients diagnosed with idiopathic hypersomnia and narcolepsy after observing the results of animal tests. It is unclear exactly when humans first ingested modafinil, but in Jouvet and Bastuji’s 1988 article they note that modafinil had been used for ‘more than three years’ with some patients (Bastuji & Jouvet, 1988, p. 699). While there was no blinding, no control group, and a relatively small sample size (42 patients in total) the results were, Billiard and Broughton (2018, p. 70) note, ‘excellent and surpassed all expectations’.

Despite these results, it was only with Jouvet’s encouragement that Lafon was convinced to continue investing in the development of modafinil. Billiard and Broughton (2018, p. 70) report that Lafon was concerned about ‘the orphan disease status of narcolepsy’, implying that he could not foresee a large enough market for the drug from which to recoup the costs of development. Thus, a connection was building between drug and indication, but market factors threatened to sever it regardless of its clinical success.

By 1987, Jouvet was bold enough to endorse military uses of modafinil. At an international NATO defence meeting, he claimed that modafinil could ‘keep an army on its feet and fighting for three days and nights with no major side-effects’ (quoted by Lyons & French, 1991, p. 432, and repeated by Billiard & Broughton, 2018, p. 70, without quotation). Why Jouvet was given space to speak at an international defence meeting is unclear, but it appears his claim was noticed.

In 1990, the French Minister of Defence requested that trials of modafinil be conducted on healthy volunteers (Commission De La Défense, 2001). The Gulf War of 1990-1991 occasioned the first use of modafinil in active military operations. The French military purchased 2,250 boxes of eight tablets of modafinil, given the label Virgyl, and distributed these among units on the ground with a short explanatory note on its use (Assemblée nationale, 2000). Despite the recommendation that this drug be used only under direct orders from senior personnel (LaGarde, 1990), decisions on its use in practice were left to regimental leaders and their field doctors (Commission De La Défense, 2001). As such, the actual prevalence of its use in these operations is unclear. The French military was not alone in its interest in modafinil. The UK Ministry of Defence similarly commissioned a study of modafinil and purchased 21,600 tablets during the Iraq war (Hansard, 2004), though actual use is again unclear.

### Negotiating modafinil to market as a drug for narcolepsy with low abuse-potential

The shift from experimental compound to authorized medical treatment is marked by the 1992 market approval of modafinil in France. Acquiring market approval brought many more actors into modafinil’s scope. The regulatory body provides a validation of the successful alignment of the compound with a diagnostic category, which in turn brings those diagnosed individuals under its remit.

Marketing efforts continued after market approval. A further two years of negotiations on pricing and reimbursement was needed before modafinil was sold on the French markets, initially available only with a prescription from public hospital neurologists and via hospital pharmacies. In 1995, this stipulation was relaxed to allow prescription by neurologists and sleep specialists in both public and private clinics.

While Lafon continued to produce modafinil for the French market, licencing agreements formed avenues of international diffusion through which modafinil made its way to more lucrative markets. Here, we focus on the largest and most aggressively pursued of these markets when, in 1993, Cephalon Inc. purchased the exclusive rights to market modafinil in the US. After the purchase, Cephalon immediately began the clinical-trial process necessary to acquire regulatory approval for the sale of modafinil to the US market. By 1996, a New Drug Application (NDA) was filed with the FDA.

Many of Cephalon’s clinical trials are published (see Billiard & Broughton, 2018, for example). What does not appear in the academic literature are the negotiations that occurred throughout the approval process regarding the labelling, abuse potential, and drug schedule classifications for modafinil. In the FDA approval package, we can see some of these negotiations taking place. This is also the first place we encounter the proprietary name, Provigil. Comparisons to existing stimulants are frequently the sites of negotiations, as highlighted previously in the patent documentation for both modafinil and adrafinil.

The FDA’s Michael Klein, who reviewed the drug abuse data, believed modafinil ‘intrinsically has considerable abuse potential’ and should be classified as a Schedule III drug (FDA, 1998, P5, p. 3). Klein cited the observation in one of the submitted studies that, when given the opportunity, monkeys would self-administer the drug and suggested that there was significant potential for more general psychological addiction (as opposed to physical dependence). It appears that, initially, Klein’s recommendations were to be followed. Three days before the NDA was due to be finalized, however, Cephalon submitted further documentation in attempts to have these decisions reversed and to have modafinil classed as a Schedule IV drug. A consultant for Cephalon, who conducted the study on abuse potential, maintained that ‘possible off-label use (for ADHD and staying awake for performance enhancement) is “not abuse, but misuse”’ (Jasiniski, in FDA, 1998, P1, p. 18).

Klein again disagreed with Cephalon’s addition of two study descriptions, highlighting different perspectives on the addictive potential of modafinil. Klein believed that one of these studies, which compared modafinil to amphetamines, was of poor quality and that the ‘conclusions reached by the sponsor are wrong, and therefore that they should not be included in labelling because they are misleading’ (FDA, 1998, P5, p. 5). Klein believed that the second study was inconclusive and, without justification, suggested that the addictive properties of modafinil are analogous to those of caffeine.

Drug scheduling is an institutional mechanism through which various actors negotiate the assignment of categories. In the criminal law system, these categories can dictate the penalties for possession, sale, or mis/use of a drug. In research, drug scheduling categories influences the ease with which researchers can access and administer a given drug, how it is stored in a laboratory or clinic, and how carefully the units must be counted at the end of each day (US Drug Enforcement Administration, 2025; Gabay, 2013). For clinicians and patients, they can act as a signpost for caution. The years of comparisons with caffeine and differentiation from amphetamine-type stimulants had been building the alignment between modafinil and the less stringent end of the drug scheduling classifications. In the end it was a success. Modafinil, after repeated back and forth between FDA representatives, Klein, and the Cephalon consultants, was classified as a Schedule IV drug, and thus officially designated as having a low potential for abuse or dependence.

Klein pushed not only for Schedule III classification, but also for an additional Abuse Potential section to be added to the Precautions section of the approved labelling. FDA representative Russell Katz recounted in a memorandum that he recommended the labelling be accepted with the FDA’s (i.e., Klein’s) proposed changes because of the impending decision deadline. The proposed changes included: ‘1) the inclusion in the Precautions section of a statement about abuse potential, 2) the absence of a description of a single dose study in normals in the Drug Abuse and Dependence section’ (FDA, 1998, P2, p. 4). This was again strongly opposed by Cephalon representatives, who called on precedents from other Schedule IV drugs that do not include such extra warning sections. The final decision was made after further consultation with Cephalon and communicated during a phone conversation in which nine Cephalon representatives and three FDA representatives participated; Klein was not amongst them. Modafinil was granted US marketing approval for the treatment of narcolepsy, classified as a Schedule IV drug, and sold without the additional abuse-potential warnings recommended by Klein.

Coincident with marketing approval for narcolepsy was modafinil’s shift to official classification as an orphan drug. The US Orphan Drug Act of 1983 had created a new legal category. It was intended to stimulate drug development by providing extra financial incentives to firms who develop drugs for conditions with low prevalence rates. As described above, concern about narcolepsy being an orphan disease precipitated reluctance by Lafon in the early stages of drug development. Narcolepsy fits the orphan description since it is estimated to affect between 0.02% and 0.05% of the population (Akintomide & Rickards, 2011). Orphan designation extended Cephalon’s exclusivity on modafinil by two years. This was valuable given that modafinil’s initial patent was due to expire in 2001, potentially allowing for the entry of generic modafinil into the market, an event that would cause profits from branded Provigil to drop dramatically.

In addition to the negotiations over classification concerning narcolepsy, Schedule IV, and orphan drug categories, the FDA documentation also revealed that Cephalon’s goals for modafinil extended beyond this. A memorandum of a conversation with Paul Nemeth of Cephalon notes that they were intending to ‘look for “a big indication” specifically ADHD …. Next year they are planning ADHD studies’ (FDA, 1998, P1 p. 18). Notably, Cephalon harboured ambitions for an ADHD indication in parallel with the assertation that off-label use for ADHD could be dismissed as merely ‘misuse’. While the attempts to have modafinil approved for use with ADHD were ultimately unsuccessful, a newly created diagnostic category would enter the fray.

### Creating new uses: How modafinil went from being an orphan to a blockbuster

In 2003, worldwide sales of Provigil totalled US$290 million (Cephalon, Inc., 2004). This was an increase of 40% over 2002, which Cephalon, Inc. (2004, p. 29) noted was:

driven by an expansion of both our sales force and marketing efforts, and …. a considerable portion of the increase in prescriptions is due to the fact that some physicians have elected to prescribe the product to treat excessive sleepiness and fatigue outside of narcolepsy.

Much greater market expansion was on the horizon, however. What was to become the largest approved indication for modafinil was not a part of the standard diagnostic manuals when the drug was first developed, nor even at the time Cephalon purchased the rights to the drug. The diagnostic category of Shift Work Sleep Disorder (SWSD), a subcategory of disorders of the circadian rhythm, was added to the Diagnostic and Statistical Manual of Mental Disorders (DSM IV) in 1994. This manual—in many contexts and particularly in the US—serves as an administrative arbitrator for what counts as a mental, psychiatric, or psychological disorder. What counts as a disorder, by extension, can determine what kinds of suffering are deemed socially legitimate claims on the services and resources of healthcare bodies, including the prescription of medication (Rosenberg, 2002, 2015).

An SWSD diagnosis, in brief, can be attributed to shift workers who have difficulty regulating sleeping and waking hours. DSM categories are regularly intertwined with our social structures and norms (Pickersgill, 2012), but SWSD is a conspicuous but relatively under-interrogated example of a category that could not exist outside of a particular set of social-economic circumstances. This categorical structure, mediated by the marketing approval of the relevant regulatory body (in the US, this is the FDA), allowed for an alliance between modafinil and sleep-deprived shift workers.

In 2003, Cephalon was granted approval from the FDA for a ‘Supplemental New Drug Application’ (SNDA) that expanded the approved indications for modafinil to include SWSD and the treatment of excessive daytime sleepiness associated with obstructive sleep apnoea (OSA). The addition of SWSD and OSA to modafinil’s approved indications drastically increased the scope of the market. Estimates of OSA prevalence range from 6% to 17% of the general adult population (Senaratna et al., 2017). Estimates of SWSD prevalence range from 10% to 20% of shift-workers (Drake et al., 2004; Fadeyi et al., 2018), with a quarter of Americans estimated to work irregular shift patterns (CDC, 2021). Together this amounts to some ~13–35 million new potential users in the US. The SNDA also provided Cephalon with three further years of exclusive rights to market the drug. Modafinil sales grew 51% in the year following the expansion of the labelling to include these conditions. Modafinil’s prescription rate for non-orphan usages was substantially and significantly higher than for its orphan designation (narcolepsy) (Kesselheim et al., 2012). However, this growth was not solely attributable to the addition of the SWSD and OSA indications.

Physicians may prescribe any FDA-approved medication for conditions not listed in its label under recommended uses; this is termed ‘off-label’ prescribing. Such off-label prescribing is discouraged because insurance companies are often unwilling to reimburse prescription costs, and because it may increase the physician’s exposure to professional malpractice liability. In practice, off-label prescribing is common, because patients often bear the prescription costs, and because the liability risk to the physician may be low if there are relevant studies, particularly if there is established precedent (Largent et al., 2009). In Cephalon’s continued search for broader markets, off-label uses were in its sights. An FDA warning letter to Cephalon cited the ‘dissemination of false or misleading promotional materials’ (FDA, 2002, p. 1), noting that modafinil was not approved as a treatment for specific symptoms such as tiredness and fatigue, and that encouraging use in this manner was illegal.

In 2008, off-label marketing charges against Cephalon led to a total of US$425 million in plea agreements (US Department of Justice, 2008). These legal cases included the charge of deliberately encouraging physicians to prescribe modafinil for conditions beyond its approved indications. Direct marketing to physicians via personal interactions and conferences, and highly paid speaking positions for physicians who regularly prescribed modafinil off-label were amongst the claims. The claim that Cephalon offered to take care of the administrative work involved in such off-label prescribing highlights the firm’s understanding of the institutional obstacles in their way.

The number of conditions for which fatigue is a recognized symptom or by-product of treatment is large, and modafinil has consequently been the subject of many clinical trials. Examples include cancer, HIV, depression/major depressive disorder, Parkinson’s disease, myotonic dystrophy, traumatic brain injury, bipolar depression, schizophrenia, cocaine addiction, fatigue in post-polio syndrome, recovery from general anaesthesia, and sleep-deprived emergency room physicians (Kumar, 2008). These might be seen to underwrite the widespread prescription of modafinil for off-label uses. One study of off-label prescription expenditure cites modafinil as second only to lidocaine patches (a local anaesthetic) (Kesselheim et al., 2012). One professional newsletter claims that 90% of modafinil prescriptions are off-label (Carlat, 2013). In the UK, the National Institute for Health and Care Excellence guidelines recognize the regular use of modafinil for the treatment of ADHD and fatigue associated with multiple sclerosis, amongst other conditions; this was reflected in a study of the ten most frequent uses for modafinil in the UK, which also included drowsiness, sedation, and chronic fatigue (M. Davies et al., 2013). Modafinil is approved in the UK to treat narcolepsy only. Beyond clinical populations, there is reportedly widespread use of modafinil for lifestyle and cognitive enhancement in various social circles (e.g., Brühl et al., 2019; Coveney, 2011, 2014; Coveney et al., 2009; Porsdam Mann et al., 2018; Sharif et al., 2021).

As sales and uses of modafinil expanded, the list of approved uses in the EU and UK shrank. Following a decision by the European Medicines Agency, the UK Medicines and Healthcare products Regulatory Agency similarly recommended in 2010 that modafinil be, ‘restricted to treat only sleepiness associated with narcolepsy, and that it should no longer be used for the treatment of excessive sleepiness associated with obstructive sleep apnoea or chronic shift work sleep disorder’ (Medicines and Healthcare products Regulatory Agency, 2010, para. 1). By this point, in 2010, worldwide sales of Provigil had risen 385.49% from 2003 to US$1.12 billion (Cephalon, Inc., 2011).

## Discussion

Our approach offers empirical insights into modafinil’s muddled development as well as theoretical insights into the roles of classification and serendipity in drug development. We followed trails of reference, examined failures alongside successes, and noted traces of uncertainty in the moments before a version of success rewrote history in its own image. The uncertainty here is an ‘affective state of individuals acting within specific sociopolitical and clinico-epistemic infrastructures’ (Pickersgill, 2020, p. 85).

Empirically, we provided an accounting of apparently small but consequential moments in modafinil’s development trajectory. Our focus was on the breadth of the search space and narrowing down of potential compounds and potential uses. Using regulatory documents, patents, and other contemporaneous sources, we showed that the nature of adrafinil was not fixed. The discovery of modafinil, a metabolite of adrafinil, produced new possibilities. Modafinil moved quickly from putative sedative to stimulant. This is not because it failed to act as a sedative; this is because its action as a sedative was contingent on considerations about dosage and risk classification. Comparisons to antidepressants and amphetamines further demonstrate uncertainty at the patent stage. Lafon moved towards use with narcolepsy. What had previously been lamented by Lafon as too small a market subsequently became an avenue for extending exclusivity rights as an orphan drug. This diagnostic category assigned to modafinil could have been otherwise, and indeed, it later was. A year after Cephalon purchased marketing rights, a new potential indication came into being. SWSD not only hugely expanded the US market for modafinil, but also legitimated the use of modafinil for issues created by one’s lifestyle and/or place in a societal structure. The list of off-label uses promoted by Cephalon was long. Modafinil became a blockbuster drug. We traced these movements from possibilities to specific outcomes, moments in time which can retrospectively be framed as unexpected success and thus serendipity.

Theoretically, we showed that the concept of serendipity can reveal assumptions hidden by linear retellings of history. It helps to locate sites of research interest, such as: where in the process uncertainties are greatest, which actors shape their closure, what is deemed successful, and who are the beneficiaries of these resolutions. Cursory attribution of serendipity to cases (i.e., absent the methodological approach we deployed above) is likely to obscure potentially useful analyses of the messy and difficult process of drug development. For example, if the initial creation of a compound and observation of a potentially useful effect were sufficient, then the ten alternative compounds specifically named and discussed in the patent documents could also be considered instances of serendipity despite being dropped from further development. We have shown that benefit or success on the part of patients, the firm, or policy-makers depends on the alignment of the compound with the interests of these groups within the institutional landscape. We have also seen that this success is not binary. While Lafon drove the creation of modafinil, for instance, it was Cephalon that reaped the greater rewards.

Taken together, the theory and empirical material presented here suggest that uncertainty permeates much of the drug development process. Additionally, viewing serendipity in this way highlights unexpected and beneficial discovery as a constrained process. Modafinil as a treatment for narcolepsy may not have been the expected outcome of the development of adrafinil, but the search was not an entirely random one. The drug developers were working within physical, cognitive, and economic spaces designed to produce psychopharmaceuticals, and they did just that. In *bounded serendipity*, the breadth of the search space is constrained and narrowing. However, such discovery marks only the early phase. The resulting compound must then be integrated into the social, clinical, regulatory and market landscapes.

For a compound to become a drug, closure is necessary on who the drug is intended for, but that closure is neither static nor final. Closure comes through the alignment of interests of a range of actors working within the constraints of these infrastructures; the processes of alignment are often costly and contested, and their outcomes are not predetermined. One way in which closure is not finite comes from shifts in the infrastructural conditions under which closure occurs. We observed this for modafinil in the opening up of new potential markets and patients through the addition of SWSD to the DSM. The realignment and thus renegotiation of the nature of the drug can also reopen and redirect closure mechanisms, as we saw through the various shifts in the predefined diagnostic categories that were linked to adrafinil and modafinil: cognitive decline, idiopathic hypersomnia, narcolepsy, etc. Serendipity is regularly ascribed to the outcomes of drug development and linked to an initial lack of knowledge about a compound and its effects on the body, as if there ought to be a self-evident relationship between drug candidate and diagnostic category (e.g., Klein, 2008). We have shown that perceptions of such direct relationships are the product of hindsight.

The creation of new markets for modafinil via encounter with emerging diagnostic categories, and through aggressive (and sometimes illegal) marketing tactics overlaps with a body of critical work on the expansion of psychiatric diagnoses and concurrent increases in prescriptions of pharmaceuticals, and their societal implications (e.g., Conrad, 2007; J. Davies, 2021; Frances, 2014; Rose, 2006). This literature examines processes of ‘medicalization’ and ‘biologization’ (Rose, 2006), which are particularly relevant to the case of modafinil and Shift Work Sleep Disorder (SWSD).^5^ These concepts describe the transformation of previously non-medical issues into medical or biological problems, often accompanied by pharmaceutical interventions. Our case study exemplifies the provisional stabilization of an artifact, illustrating how diagnostic categories can be leveraged to expand pharmaceutical markets.

Lastly, there are two important implications for policy. First, perceptions of randomness—and serendipitously discoverable direct relationships between compound and use—risks overlooking the scope for policy and strategy to play an active role in shaping malleable outcomes. Conversely, understating uncertainty risks disregarding all the failures and dead ends that plague development; the long and arduous work that is done after discovery.

Second, applying our insights to drug repurposing, we may expect that drugs which are known to act on, for example, the central nervous system (CNS), will be likely candidates for alternative CNS diagnoses. These potential connections to indications should, however, not be expected to simply show themselves. Although repurposing enters the process when some uncertainties are already resolved, the choice of compound and its use should develop in parallel, and should be expected to be effortful, time consuming, and likely expensive.

Further research may help to develop understanding of contingent classification and the bounded nature of serendipity in drug development. Our study calls for a closer examination of the interactions between diagnostic categories, policy and regulation in shaping drug trajectories, the impact of market forces, and the potential for drug repurposing in the development process.

## Notes

1. The patents mention testing on both rats and mice. However, given that mice are used to test toxicity and lethal dosage, it is likely that testing in mice occurred prior to the behavioural testing that took place with rats.

2. We use the terms adrafinil and modafinil to aid readability, rather than to pre-empt the outcomes of CRL40,028 and CRL40,476. The former labels are indicative of the results of the work that we are discussing in these sections; the latter labels would be more consistent with uncertainty but more cumbersome for the reader.

3. Although there are inconsistencies in the approval date in the literature, 1981 is the date stated in the *communique* from the Agence française de sécurité sanitaire et des produits de santé, the French regulator, regarding the withdrawal of approval (AFSSAPS, 2011).

4. For example, Lafon was granted US patent 4209523, which covers a set of related compounds: ‘acetohydroxamic acids’, which were claimed to be useful ‘in therapy treating the central nervous system (CNS), especially as psychotropic agents’. In this patent are descriptions of CRL40,385, which Chariot et al. (1987) later refer to as a ‘parent compound’ of modafinil and adrafinil. Another patent was granted in the UK in 1980, which covers CRL40,412, another acetamide derivative (although more chemically removed from modafinil/adrafinil than that mentioned above) that is described as having effects consistent with a sedative.

5. While modafinil was already approved for a pre-existing indication, there is a sense in which it paralleled Conrad’s (2007, p. 29) description of testosterone as ‘a drug in search of a disease to treat’. We thank one of the reviewers for highlighting this parallel.
